# Function of *Akkermansia muciniphila* in type 2 diabetes and related diseases

**DOI:** 10.3389/fmicb.2023.1172400

**Published:** 2023-06-15

**Authors:** Jinjie Li, Ge Yang, Qihe Zhang, Zhuo Liu, Xin Jiang, Ying Xin

**Affiliations:** ^1^Jilin Provincial Key Laboratory of Radiation Oncology & Therapy, The First Hospital of Jilin University, Changchun, China; ^2^Key Laboratory of Pathobiology, Ministry of Education, College of Basic Medical Science, Jilin University, Changchun, China; ^3^Department of Gastrointestinal Colorectal and Anal Surgery, China-Japan Union Hospital of Jilin University, Changchun, China; ^4^Department of Radiation Oncology, The First Hospital of Jilin University, Changchun, China; ^5^NHC Key Laboratory of Radiobiology, School of Public Health, Jilin University, Changchun, China

**Keywords:** *Akkermansia muciniphila*, type 2 diabetes mellitus, gut microbiota, metabolic diseases, endotoxemia, prebiotics

## Abstract

The prevalence of type 2 diabetes (T2D) is increasing worldwide, with many patients developing long-term complications that affect their cardiovascular, urinary, alimentary, and other systems. A growing body of literature has reported the crucial role of gut microbiota in metabolic diseases, one of which, *Akkermansia muciniphila,* is considered the “next-generation probiotic” for alleviating metabolic disorders and the inflammatory response. Although extensive research has been conducted on *A. muciniphila*, none has summarized its regulation in T2D. Hence, this review provides an overview of the effects and multifaceted mechanisms of *A. muciniphila* on T2D and related diseases, including improving metabolism, alleviating inflammation, enhancing intestinal barrier function, and maintaining microbiota homeostasis. Furthermore, this review summarizes dietary strategies for increasing intestinal *A. muciniphila* abundance and effective gastrointestinal delivery.

## 1. Introduction

Diabetes mellitus (DM) refers to a group of metabolic disorders characterized by hyperglycemia, insulin resistance, obesity, and low-grade inflammation (Zimmet et al., 2001). The prevalence of diabetes is increasing at an alarming rate worldwide. In 2017, the International Diabetes Federation estimated that there were 451 million patients with diabetes and 374 million people with impaired glucose tolerance worldwide (Cho et al., 2018). If there is no radical therapy to reverse this expansion, the total number of people with diabetes could increase to 693 million by 2045 (Cho et al., 2018). Type 2 diabetes (T2D) would account for more than 90% of cases in this population (Chatterjee et al., 2017). T2D is a heterogeneous disease, and various factors may contribute to its development, such as unhealthy lifestyle and diet, genetic heritage, aging, and environmental factors (Chatterjee et al., 2017). Due to the long duration of diabetes, T2D patients have higher risks of macrovascular and microvascular damage, as well as other severe complications, such as diabetic nephropathy, diabetic cardiomyopathy, non-alcoholic fatty liver disease (NAFLD), diabetic retinopathy, cognitive impairment, that cause mortality (Gregg et al., 2014). The high incidence of T2D indicates that previous preventive and therapeutic approaches are ineffective. Recent studies have examined the effects of gut microbiota on metabolic diseases, including T2D (Cani, 2018; Gurung et al., 2020). Furthermore, diabetes treatments that target the gut microbiota have become a growing area of investigation.

In the human body, microbial cells outnumber human cells by approximately 10-fold and contribute significantly to host metabolism and immunity (Cani, 2018). As per observational studies in humans, the genera of opportunistic pathogens (i.e., *Ruminococcus*, *Fusobacterium*, and *Blautia*) were enriched in T2D patients, while the genera *Bifidobacterium*, *Bacteroides*, *Faecalibacterium*, and *A. muciniphila* were negatively associated with T2D (Gurung et al., 2020). *Akkermansia muciniphila* is a gram-negative, strictly anaerobic, oval-shaped, non-spore-forming mucin-degrading bacterium that belongs to the division *Verrucomicrobia* and was first isolated from the feces of healthy adults in 2004 through the use of mucin as the only source of carbon and nitrogen (Derrien et al., 2004; Ouwerkerk et al., 2016; Machado et al., 2020). *A. muciniphila* mostly colonizes the intestinal tract and accounts for approximately 1–3% of the total microbiota (Derrien et al., 2008). The bacteria have been detected in fecal samples of healthy adults of all ages, at levels ranging from 5.00 to 8.80 log cells/g, but their population is reduced among most elderly individuals (Collado et al., 2007). Research on *Akkermansia* is of growing interest, many animal experiments have determined the positive effects of *A. muciniphila* supplementation on diabetes and related diseases (Cani and de Vos, 2017). Similarly, the administration of *A. muciniphila* improved the metabolic parameters of obese patients (Depommier et al., 2019). However, the exact mechanism underlying these positive effects remains unclear. Herein, we summarize research progress on *A. muciniphila* and describe its role in T2D and related diseases, from its mechanism of action to its therapeutic application.

## 2. The diversity of *Akkermansia muciniphila* strains

In light of the known benefits of *A. muciniphila*, researchers have attempted to determine its genome and the functions of the encoded proteins. *A. muciniphila* belongs to the phylum Verrucomicrobia and displays significant diversity among the different strains (van Passel et al., 2011). Guo et al. (2017) constructed the genomes of 39 *A. muciniphila* strains isolated from adult humans and laboratory mice and identified three major phylogroups using maximum likelihood phylogenetic analysis. Later, Kirmiz et al. (2020) reclassified *A. muciniphila* isolates into four species-level phylogroups based on a predecessor (defined as AmI, AmII, AmIII, AmIV). Becken et al. (2021) suggested that the AmI phylogroup can be phylogenetically divided into two subclades, AmIa and AmIb. At present, the commonly used strain in most researches is *A. muciniphila* Muc^T^ (=ATCC BAA-835^T^ = CIP 107961^T^), belonging to AmIa. Differences among these four phylogroups include intestinal abundance, physicochemical properties, metabolic characteristics, and immune activation capacity. In a study of 1617 human fecal samples (Karcher et al., 2021), AmI accounted for the highest proportion (47%), followed by AmII and AmIII (27 and 24%, respectively). AmII and AmIII are currently only observed in humans, whereas AmI and AmIV are common in humans and mice in different proportions. Another metagenomic analysis showed that AmIV is mainly distributed in western populations, while AmIII is mainly found in China (Lv et al., 2022).

Genome sequencing has revealed the genes differentially expressed among the four phylogroups, AmI, AmII, AmIII, and AmIV. According to the Kyoto Encyclopedia of Genes and Genomes (KEGG) pathway analysis, genes in the *A. muciniphila* genome encode proteins involved in membrane transport, drug resistance, and various metabolic activities (Guo et al., 2017). Metabolism-related genes have attracted attention due to their function in improving host metabolism. Several studies have reported differential expression of genes related to glycolipid metabolism and amino acid metabolism among *A. muciniphila* phylogroups. A principal component analysis of the KEGG pathway showed greater similarities between the AmII and AmIII genomes, whereas AmI genomes differed significantly. Specifically, all four *A. muciniphila* phylogroups encoded glycoside hydrolases (GHs) and glycosyltransferases (GTs), but the copy numbers of the GH and GT families differed from one another (Becken et al., 2021; Luna et al., 2022). The genomes of the AmI phylogroup contained fewer genes encoding fucosidases (GH29 and GH95) than the other phylogroups that potentially act on the terminal fucose residues that decorate mucin and human milk oligosaccharides (Wu et al., 2021). Notably, AmIb had fewer GH29 genes than AmIa and no GH18 genes (Luna et al., 2022), and galactosidase GH110 was hardly expressed in AmIV (Becken et al., 2021). Similarly, the four phylogroups varied in the proportions of GT2 and GT4, suggesting that they differ in their carbohydrate metabolic levels, especially the ability to synthesize polysaccharides (Karcher et al., 2021). Furthermore, cobalamin (vitamin B_12_) is an essential cofactor for short-chain fatty acid metabolism. Kirmiz et al. (2020) found that vitamin B_12_ synthesis genes were absent in AmI. This finding suggests that the vitamin B_12_ biosynthesis genes were originally present in all strains and were lost by some isolates in the human gut (Karcher et al., 2021). Considering that several intestinal microbes require vitamin B_12_ as a metabolic substrate (Degnan et al., 2014), these genetic losses may explain the diversity of *A. muciniphila* and its interactions with the host and other intestinal microbes. Genes for assimilatory sulfate reduction (ASR) were absent in AmII and AmIV. This resulted in the absence of hydrogen sulfide, a critical substrate for cysteine and methionine synthesis, and led to a low growth rate in the mucin medium (Becken et al., 2021). Additionally, AmIV’s high sensitivity to oxygen may be attributed to a deficiency in siderophores (Becken et al., 2021). These results explain the observed differences in the distribution and oligosaccharide utilization of *A. muciniphila* phylogroups (Luna et al., 2022), and may also explain the reason for AmI and AmII to have different therapeutic efficacies in alleviating metabolic syndrome (Deng et al., 2020).

The relationship between *A. muciniphila* and colitis is controversial. Several lines of evidence have shown that *A. muciniphila* administration can ameliorate inflammatory bowel diseases (IBD) or delay colitis-associated tumorigenesis in mice (Wang et al., 2020; Wade and Su, 2021), whereas certain oral treatments for colitis have enriched the abundance of *A. muciniphila* (Ke et al., 2021). While some studies have suggested that *A. muciniphila* aggravates IBD (Ganesh et al., 2013), Ring et al. (2019) proposed that ingestion of the *A. muciniphila* strain ATCC BAA-835 did not exacerbate intestinal inflammation in interleukin (IL)-10-deficient mice. Notably, Cekanaviciute et al. (2017) found increased *A. muciniphila* levels in MS patients. The investigators hypothesized that this difference might be due to strain specificity (Ring et al., 2019), and Liu’s findings substantiated their hypothesis (Liu Q et al., 2021). *A. muciniphila* displayed strain-dependent effects on ulcerative colitis in mice. AmII and AmIV exerted stronger immune activation than AmI in HEK-TLR reporter cell lines (Becken et al., 2021). In summary, strain specificity of the gut microbiota is vital for phenotypic studies (Geva-Zatorsky et al., 2017), and *A. muciniphila* also exhibited strong strain specificity. Most current studies have focused on the strain ATCC BAA-835^T^ (=CIP 107961^T^), and further investigation should be conducted to gain deeper insights into the interactions between the host and *A. muciniphila*.

## 3. The role of *Akkermansia muciniphila* in T2D and related diseases

**Figure 1:**
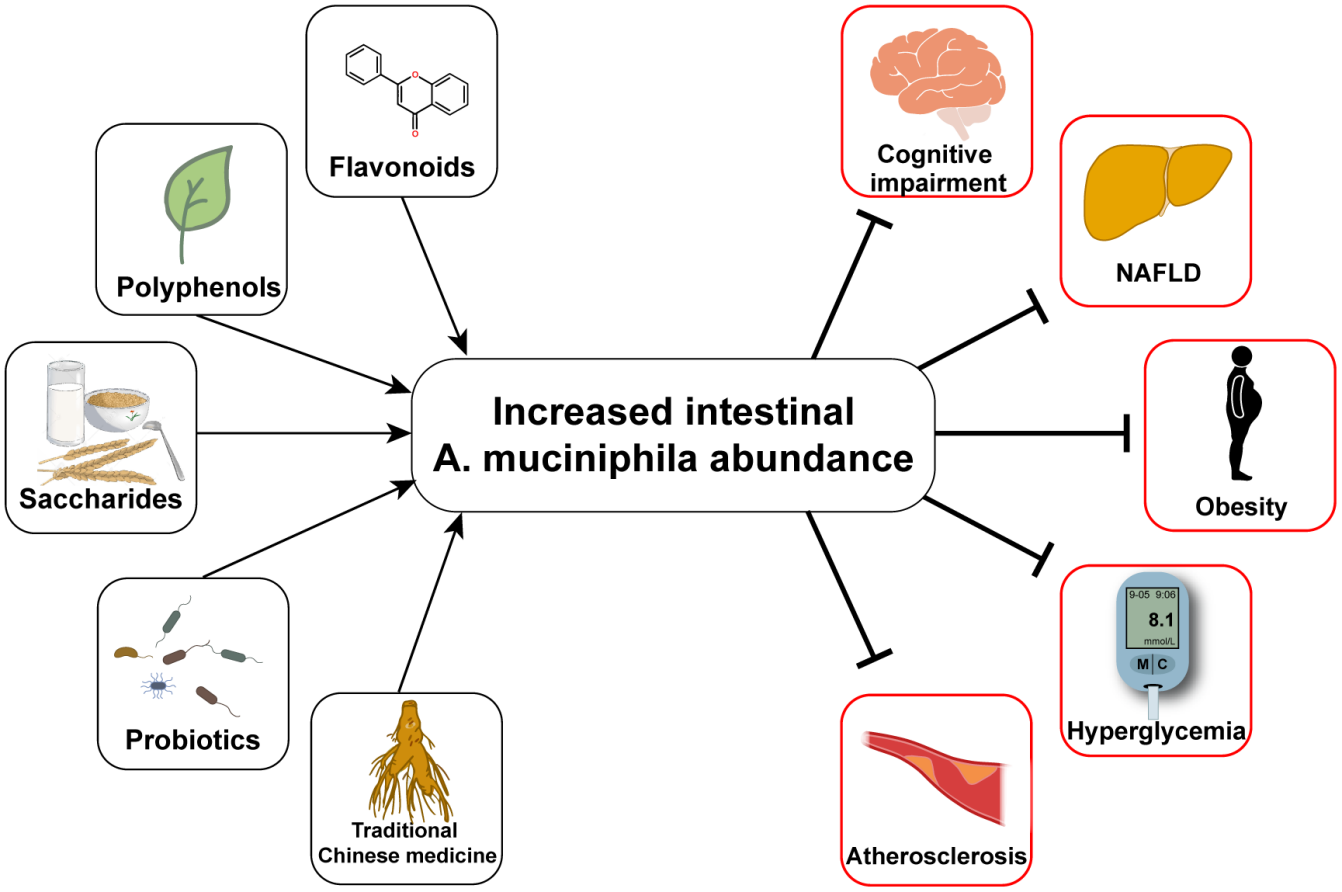
Oral measures to improve the abundance of *Akkermansia muciniphila* and the consequent improvement of T2D and its complications.

Typical manifestations of diabetes include obesity, insulin resistance, impaired glucose tolerance, abnormal lipid metabolism, and low-grade systemic inflammation (Weir and Bonner-Weir, 2004). Furthermore, human gut microbial sequencing revealed a decline in *A. muciniphila* abundance in T2D patients as early as 10 years ago (Zhang et al., 2013). Numerous studies have also found reduced *A. muciniphila* levels associated with not only diabetes (Yassour et al., 2016; Chelakkot et al., 2018; Medina-Vera et al., 2019), but also its related diseases, including hyperlipidemia (Yu et al., 2021), non-alcoholic fatty liver disease (Kim et al., 2020; Zhang et al., 2022), and chronic kidney disease (Hsu et al., 2020). Since Everard et al. (2013) discovered the effect of *A. muciniphila* on alleviating metabolic disorders, researchers have conducted many investigations on this next-generation microorganism, as summarized in Table 1. Several cohort studies have revealed the beneficial effects of *A. muciniphila* on glucose and lipid metabolism and inflammatory responses in humans (Depommier et al., 2019; Perraudeau et al., 2020). Furthermore, a few animal experiments have also demonstrated multiple benefits of *A. muciniphila* in liver disease, cardiovascular disease, cognitive impairment, and aging (Grajeda-Iglesias et al., 2021). The diseases related to *A. muciniphila* and several classes of oral supplements, including polyphenols, saccharides, and flavonoids, that increase the abundance of *A. muciniphila* are outlined in Figure 1. Furthermore, pasteurized *A. muciniphila* has been confirmed to be safe and was approved for use by the European Food Safety Authority in 2021 [Efsa Panel on Nutrition, Novel Foods and Food Allergens (NDA) et al., 2021].

**Table 1:** The efficacy of oral *Akkermansia muciniphila* in T2D and related diseases.

Disease	Efficacy	Mechanism	References
Type 2 diabetes (hyperglycemia)	Blood glucose, insulin resistance, glycosylated hemoglobin↓ Insulin secretion, glucose tolerance ↑	GLP-1↑ FGF15/19↑	Depommier et al. (2019, 2020), Everard et al. (2013), Hanninen et al. (2018), Org et al. (2015), Perraudeau et al. (2020), Plovier et al. (2017), Yang et al. (2020), Zhao et al. (2017)
Type 2 diabetes (low-grade inflammation)	Metabolic endotoxemia, pro-inflammatory factors↓ Tight junction, intestinal barrier function, anti-inflammatory factors, goblet cells, Treg cells↑	Wnt3↑ AhR↑ Mucin2↑	Depommier et al. (2019), Everard et al. (2013), Grander et al. (2018), Plovier et al. (2017), Yoon et al. (2021)
Hyperlipidemia and obesity	Body weight, triglyceride, total cholesterol, chylomicrons, fat mass↓ Adipose thermogenesis↑	UCP1↑ FGF15/19↑ PLIN2↓	Deng et al. (2020), Katiraei et al. (2020), Shen et al. (2016)
Liver disease	Alanine aminotransferase, alanine transaminase, hepatic apoptosis, liver steatosis, adipogenesis, myeloperoxidase-positive neutrophils↓ Intestinal barrier function, lipid transportation and oxidation, hepatic L-aspartate↑	LKB1↑ FGF15/19↑	Everard et al. (2019), Juarez-Fernandez et al. (2021), Kim et al. (2020), Rao et al. (2021), Wu et al. (2017), Xia et al. (2022)
Cardiovascular disease	Atherosclerotic lesion formation, metabolic endotoxemia↓	Gut barrier function↑	Li et al. (2016)
Cognitive impairment	Hippocampal function, cognitive deficits, impaired spatial working memory, novel object recognition↓ Neuronal development and synapse plasticity↑	Unknown	Higarza et al. (2021), Wu et al. (2020), Yang et al. (2019)

Research subject: Depommier et al. (2019) and Perraudeau et al. (2020) chose humans as the research subject, the rest chose mice as the research subject. Adopted *A. muciniphila* strains: Yang et al. (2020) and Wu et al. (2020) used strains newly isolated from human fecal samples; Deng et al. (2020) chose AmI (GP01) and AmII (GP25) separately, the rest used *A. muciniphila* Muc^T^ (=ATCC BAA-835 T = CIP 107961 T). Plovier et al. (2017) and Depommier et al. (2019, 2020) also used pasteurized *A. muciniphila*. Oral administration: By gavage or added *A. muciniphila* to sterile drinking water. GLP-1, glucagon-like peptide-1; FGF15/19, fibroblast growth factor 15/19; AhR, aryl hydrocarbon receptor; UCP1, uncoupling protein 1; PLIN2, perilipin2, LKB1, liver kinase B1.

## 4. Regulatory mechanisms of *Akkermansia muciniphila* in T2D and related diseases

### 4.1. *Akkermansia muciniphila* treatment improves host metabolism

Insulin resistance and metabolic syndrome are the most prominent characteristics of T2D, and *A. muciniphila* alleviates metabolic syndrome by improving glucose, lipid, and bile acid metabolism. Glucagon-like peptide-1 (GLP-1) is a pleiotropic hormone that has a broad role in metabolism regulation, including the stimulation of insulin secretion and appetite suppression (Muller et al., 2019), both of which contribute to diabetes treatment. Propionate, an *A. muciniphila* metabolite, has been shown to stimulate GLP-1 secretion (Chambers et al., 2015; Psichas et al., 2015). Furthermore, Yoon et al. (2021) proposed a new GLP-1 activator, intercellular adhesion molecule 2 (ICAM-2), which is an immune cell integrin (Iannacone, 2016). ICAM-2, when directly combined with *A. muciniphila*-derived protein P9, induced GLP-1 secretion and ameliorated hyperglycemia. Additionally, Xia et al. (2022) observed the activation of the phosphatidylinositol 3-kinase (PI3K)-Akt pathway in mouse liver upon gavage of *A. muciniphila*. Notably, PI3K-Akt signaling plays an important role in glucose and lipid metabolism (Huang et al., 2018).

Scientists have reported that *A. muciniphila* regulates lipid metabolism in many ways in mice and organoids (Lukovac et al., 2014; Katiraei et al., 2020), mainly in the liver, small intestine, and adipose tissue. In the intestine, *A. muciniphila* degrades mucin and produces a variety of bioactive metabolites, such as polysaccharides, short-chain fatty acids (SCFA), and indole derivatives. Propionate modulates the expression of genes involved in fatty acid uptake and oxidation, such as Fiaf, Gpr43, peroxisome proliferator-activated receptor gamma (PPARγ), and histone deacetylases (HDACs) (Lukovac et al., 2014; Yoon et al., 2021). In mouse adipose tissue, *A. muciniphila* administration reduces white adipose tissue (WAT) volume and enhances thermogenesis by upregulating the uncoupling protein 1 (Ucp1)(Deng et al., 2020; Yoon et al., 2021), and downregulating the expression of lipid-droplet regulator associated protein perilipin2 (Depommier et al., 2020; Juarez-Fernandez et al., 2021). Increased colonization of *A. muciniphila* regulated a network of genes involved in lipid transportation and oxidation in hepatocytes. Shen et al. (2016) found that the reduction in hepatic low-density lipoprotein (LDL) receptor and apolipoprotein E levels in response to treatment with *A. muciniphila* contributed to the clearance of the plasma triglyceride-rich lipoprotein and chylomicron remnants in mice. Additionally, both Zhao et al. (2017) and Rao et al. (2021) reported altered expression of fatty acid translocase (FAT) with different trends. Notably, Rao et al. identified several significant differential metabolites in the mouse liver between groups with or without the administration of *A. muciniphila*. L-aspartate displayed a high fold-change (Rao et al., 2021). Additional research has shown that *A. muciniphila* facilitates L-aspartate transportation in the gut-liver axis for the activation of the liver kinase B1 (LKB1)-AMPK pathway and increases lipid oxidation, thereby ameliorating liver steatosis (Rao et al., 2021).

Bile acids (BAs) are steroid molecules that are synthesized from cholesterol in the liver and act as metabolic regulators via the nuclear farnesoid X receptor (FXR) and takeda G protein-coupled receptor 5 (TGR5)(Sonne, 2021). After the administration of the synbiotic of *A. muciniphila* and quercetin, plasma primary bile acids to secondary bile acids ratio was significantly increased in mice (Juarez-Fernandez et al., 2021). Besides, the elevated ratio of free to conjugated BA (CA/GCA + TCA) denoted the enhanced activity of bile salt hydrolase (BSH) (Juarez-Fernandez et al., 2021), which promoted the deconjugation of conjugated BA and conversion to free BA. However, BSH activity has been detected in various other gastrointestinal bacteria but not in *A. muciniphila* (Horackova et al., 2018). The increased BSH activity was probably due to the regulatory action of *A. muciniphila* on other intestinal bacteria. An observational study discovered a positive relationship between the proportion of primary and conjugated BAs with NAFLD (Puri et al., 2018); therefore, the changes in BAs induced by synbiotic treatment might have a paradoxical role in NAFLD.

Zhang J. et al. (2021) reported that treatment with *A. muciniphila* promoted insulin secretion by limiting the availability of 3β-chenodeoxycholic acid (βCDCA) in mice. CDCA was shown to be a high-affinity FXR agonist synthesized in the liver (Makishima et al., 1999), whereas βCDCA was derived from CDCA and had different configurations and functions. CDCA-mediated FXR stimulation occurs in ileal enterocytes and hepatocytes and mediates the expression of fibroblast growth factor 15/19 (FGF 15/19) and small heterodimer protein (SHP) (Sonne, 2021). The FXR-FGF15/19 and FXR-SHP pathways are critical for maintaining metabolic homeostasis. Interestingly, FXR agonists and inhibitors displayed beneficial effects on metabolic disorders, requiring more in-depth research. Zhang J. et al. (2021) observed that treatment with *A. muciniphila* limited βCDCA synthesis in mice, thereby triggering FGF19 signaling and insulin secretion. Of note, the content of CDCA is low in mice, but the authors also found that serum βCDCA negatively correlated with the relative abundance of *A. muciniphila* in clinical studies.

**Figure 2:**
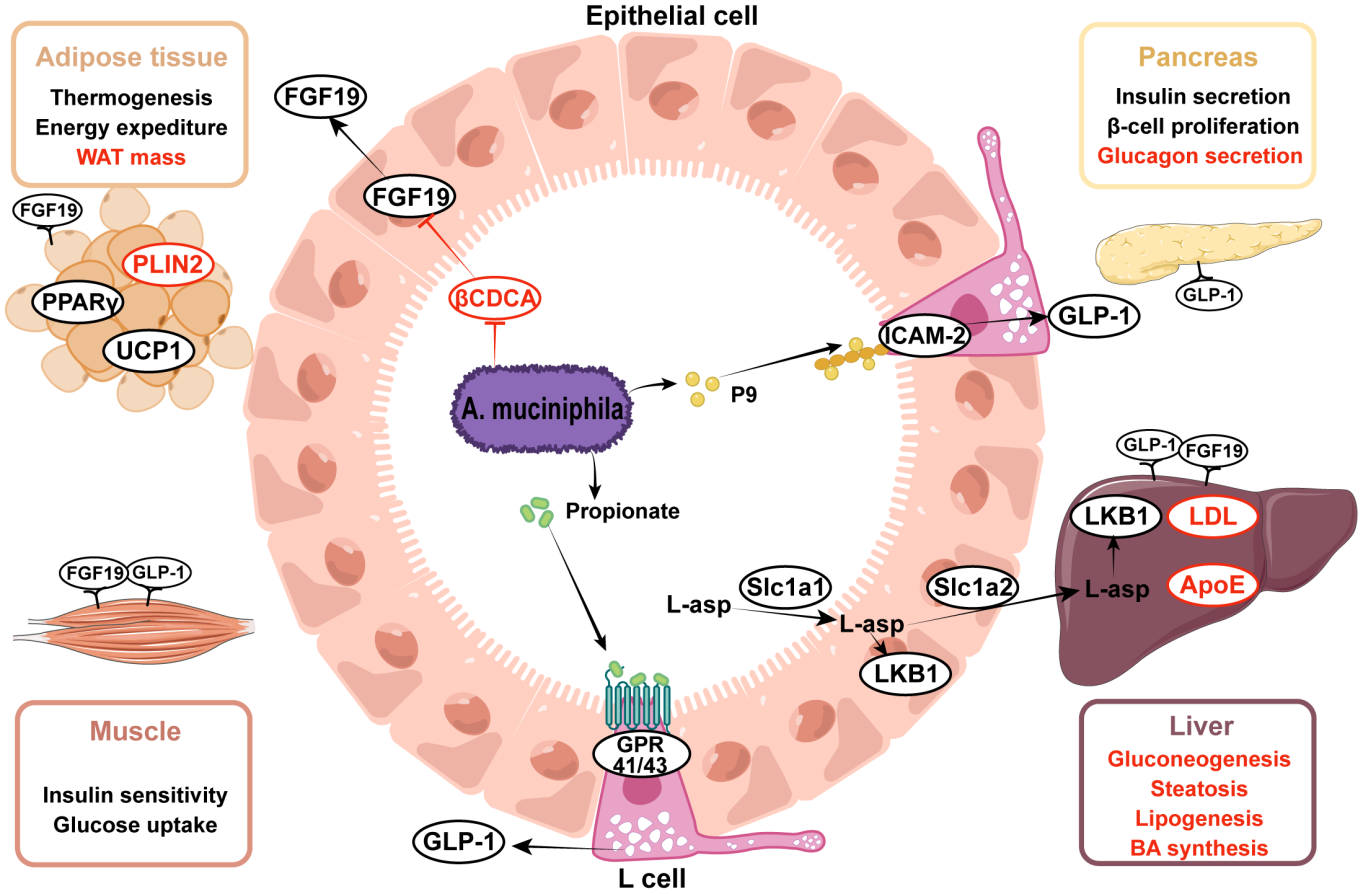
Effects of *Akkermansia muciniphila* and its derived parts on ameliorating metabolic disorders. (1) *A. muciniphila*-derived protein P9 directly combined with ICAM-2 and activated the GLP-1 secretion in intestinal L cells, propionate was the metabolite of *A. muciniphila* and also activated the GLP-1, thereby enhancing insulin secretion and alleviating hyperglycemia. (2) *A. muciniphila* modulated the expression of genes involved in lipid metabolism, including PPARγ, PLIN2, UCP1, ApoE, LDL. (3) *A. muciniphila* facilitated the transportation of L-aspartate from gut to liver, L-aspartate upregulated the LKB1-AMPK pathway and ameliorated liver steatosis. (4) *A. muciniphila* decreased the level of βCDCA, which was one of the FXR inhibitors, thus increasing the intestinal FGF15/19 secretion. FGF15/19 acted on multiple tissues, improved hyperlipidemia and liver steatosis. Upward aspects are indicated in black, downward aspects are indicated in red. ICAM-2, intercellular adhesion molecule 2; GLP-1, glucagon-like peptide-1; FGF15/19, fibroblast growth factor 15/19; βCDCA, 3β-chenodeoxycholic acid; FXR, farnesoid X receptor; L-asp., L-aspartate; LKB1, liver kinase B1; HDAC, histone deacetylases; UCP1, uncoupling protein 1; PLIN2, perilipin2; PPARγ, peroxisome proliferator-activated receptor gamma; LDL, low-density lipoprotein; ApoE, apolipoprotein E.

These results illustrate that *A. muciniphila* regulates the host metabolism of glucose, lipids, and bile acids through multiple signaling pathways, thereby alleviating insulin resistance, hyperglycemia, and lipid deposition in various organs, as outlined in Figure 2.

### 4.2. *Akkermansia muciniphila* treatment protects the host from endotoxemia

Previous research has found that patients with diabetes were more susceptible to metabolic endotoxemia than healthy people because of the increased infiltration of lipopolysaccharide (LPS) from the intestine into circulation. Notably, LPS infiltration exacerbated insulin resistance and diabetes and led to various diabetic complications (Cani et al., 2007; Gomes et al., 2017). Improved gut permeability and reduced LPS infiltration after treatment with *A. muciniphila* have been confirmed in many animal studies (Li et al., 2016; Zhao et al., 2017; Shi et al., 2021; Guo et al., 2022). The intact gut barrier consists of three main interconnected layers: the mucus, gut epithelial, and inner mucosal immune layers. Immunohistochemical staining of colon sections has shown that the colonic mucus layer is significantly thicker in *A. muciniphila*-treated mice than in vehicle-treated mice (Hanninen et al., 2018; van der Lugt et al., 2019), this may be due to the increased goblet cell count and reduced crypt depth. Furthermore, as a chemical barrier, the mucus layer contains mucin, antimicrobial peptides (AMP), immune cytokines, and digestive secretions. Mucin2 (Muc2) is a basic gel-forming mucin present in the mucus barrier (Johansson et al., 2011). Both *in vivo* and *in vitro* experiments have reported that the elevated Muc2 gene expression upon treatment with *A. muciniphila* corresponds with morphological changes and strengthens mucus barrier function (Ganesh et al., 2016; Ke et al., 2021).

The second layer of the gut barrier is composed of a monolayer of intestinal epithelial cells and intercellular tight junction (TJ) proteins, with a scattered distribution of functionally specialized cells (e.g., Paneth, goblet, and enteroendocrine cells) (Ghosh et al., 2021). After treating chickens with *A. muciniphila*, Zhu et al. (2020) found the Wnt/β-catenin signaling pathway to be activated in the intestinal stem cells, which is vital for developing and renewing the intestinal epithelium. Kim et al. (2021) further revealed that Wnt signaling activation was mediated by Paneth cells that secreted Wnt3 in the crypt, thereby accelerating intestinal stem cell proliferation. *A. muciniphila* increase the expression of tight junction proteins in mice, including zonula occludens-1 (ZO-1), occludin, claudin-3, junctional adhesion molecules-3 (jam-3) (Li et al., 2016; Wade and Su, 2021). These proteins form a complex of intercellular junctions that act as an intercellular barrier that separates luminal contents from the subepithelial interstitium (Slifer and Blikslager, 2020). However, the change in claudin-2 expression remains controversial: Liu J. H. et al. (2020) reported increased intestinal expression of claudin-2 in *A. muciniphila*-treated mice, while several studies found claudin-2 expression to be inhibited (van der Lugt et al., 2019; Wade and Su, 2021). Notably, all these studies confirmed the positive effects of *A. muciniphila* on intestinal permeability and inflammation. Claudin-2 is a pore-forming claudin that functions as a paracellular channel and increases epithelial layer permeability (Günzel and Yu, 2013). Claudin-2 expression is more likely to have declined considering the improvement in barrier function, but the precise mechanism through which *A. muciniphila* regulates claudin-2 expression remains to be elucidated.

The last line of defense consists of numerous immunocytes and immunoreactive substances. Several animal experiments have reported that *A. muciniphila* supplementation induced variations in immune cell composition (Katiraei et al., 2020), and enhanced peritoneal leukocyte functions, including chemotactic activity, phagocytic efficacy, natural killer activity, and lymphoproliferative capacity (Cerro et al., 2022). The known reason may be that *A. muciniphila* alleviates the intestinal barrier dysfunction through TLR2 activation (Shi et al., 2022), or maintains the function of intestinal barrier by activating alpha kinase 1 and downstream Nuclear Factor-Kappa B (Martin-Gallausiaux et al., 2022). Some anti-inflammatory cytokines, such as IL-10 (Ottman et al., 2017b; Katiraei et al., 2020; Yaghoubfar et al., 2020), α-tocopherol, and β-sitosterol (Zhao et al., 2017), positively correlate with *A. muciniphila* abundance. In contrast, the levels of several pro-inflammatory factors, such as IL-1β, IL-6, IL-8, and leptin (Ke et al., 2021; Cerro et al., 2022), displayed a downward trend. Ottman et al. (2017b) calculated the ratio of the pro-inflammatory factor TNF-α levels to the anti-inflammatory cytokine IL-10 levels in mice, which is an important parameter for estimating the inflammatory regulation capacity of gut microbiota. Compared to *F. prausnitzii* and *L. plantarum*, *A. muciniphila* showed the lowest ratio, implying it has high anti-inflammatory activity. Aryl hydrocarbon receptor (AhR) is a transcriptional regulator widely expressed in the intestinal epithelium and mediates antimicrobial immunity. Furthermore, impaired AhR agonist activity and lower AhR ligand concentrations are associated with metabolic disorders and intestinal barrier dysfunction (Natividad et al., 2018). AhR ligands include several indole derivatives, such as indoleacetic acid (IAA), indole-3-ethanol, and indole acrylic acid (IA), produced by tryptophan (Trp) metabolism (Gu et al., 2021; Shi et al., 2021). Gu et al. (2021) noted that *A. muciniphila* raised the plasma concentrations of IAA and IA in mice, both of which combine with AhR and activate its downstream signals. This was evidenced by increased CYP1A1, interleukin-10 (IL-10), and IL-22 levels that consequently alleviate metabolic syndrome and inflammation.

**Figure 3:**
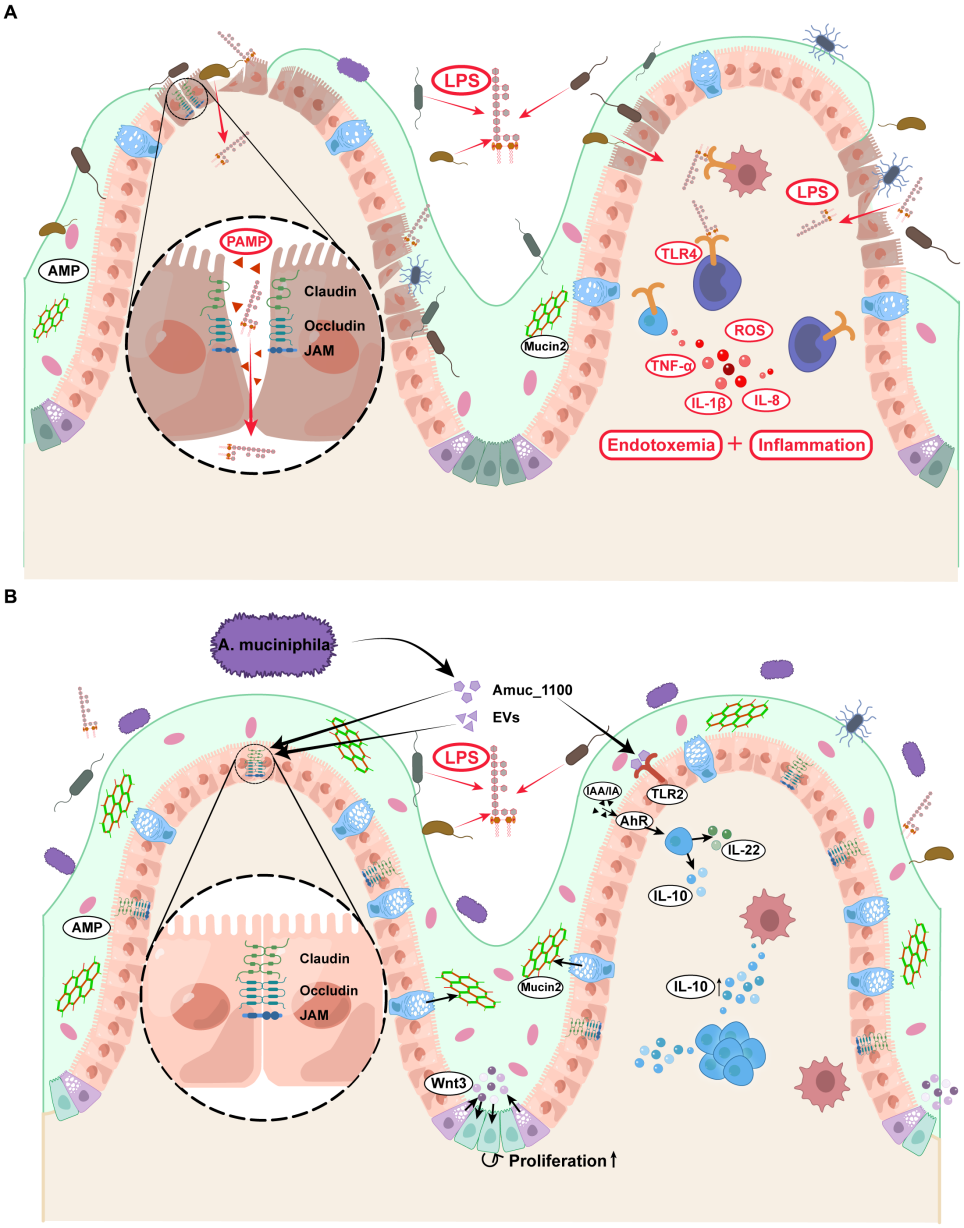
Effects of *Akkermansia muciniphila* and its derivatives on the enhancement of intestinal barrier and the prevention of endotoxemia. **(A)** Diabetic patients displayed increased gut permeability and LPS absorption, leading to the accumulation of inflammatory factors and metabolic endotoxemia. **(B)** (1) *A. muciniphila* and its derivatives directly activated the intestinal TLR2 and AhR, thereby regulating the ratio of pro−/anti-inflammatory factors. (2) *A. muciniphila* and its derivatives strengthened the function of tight junction between intestinal epithelial cells and promoted the intestinal stem cell proliferation. (3) *A. muciniphila* increased the Mucin2 secretion from the goblet cells, which was a major protein in the mucus layer. *A. muciniphila* consolidated the above three layers of intestinal barrier and prevented the LPS penetration. EVs, extracellular vesicles; LPS, lipopolysaccharide; TLR2, Toll-like receptor 2; AhR, aryl hydrocarbon receptor; IL-10, interleukin-10; IL-22, interleukin-22; AMP, antimicrobial peptide.

The above literature provides important insights into the mechanism by which *A. muciniphila* consolidates the intestinal barrier and regulates the balance of pro−/anti-inflammatory factors, thereby protecting the host from endotoxemia and metabolic disorders (Figure 3). However, the role of *A. muciniphila* in colitis remains controversial. Notably, *A. muciniphila* cannot be completely classified as anti-or pro-inflammatory, but undoubtedly, plays an essential role in endotoxemia and diabetes-related chronic inflammation (Everard et al., 2013; Plovier et al., 2017; Hanninen et al., 2018).

### 4.3. *Akkermansia muciniphila* regulates intestinal microbial homeostasis

A wide variety of microbiota colonize the human body and constitute a complex ecosystem. Maintaining the homeostasis of this ecosystem is essential for human health. Drastic changes in the composition or distribution of the microbiota result in intestinal microbial dysbiosis, which leads to various metabolic and inflammatory diseases. Several studies, having benefited from the advent of metagenomic sequencing, described the different compositions of gut microbiota between patients with T2D and healthy individuals (Qin et al., 2010). Generally, T2D has been correlated with increased levels of opportunistic pathogens (e.g., *Ruminococcus*, *Fusobacterium*, and *Blautia*) and decreased levels of SCFA-producing bacteria (e.g., *Bifidobacterium*, *Bacteroides*, *Faecalibacterium*, *A. muciniphila*, and *Roseburia*) (Gurung et al., 2020). The lower intestinal abundance of *A. muciniphila* has been demonstrated in both T2D mice and patients (Everard et al., 2013; Yassour et al., 2016). Although there is no exact definition of a healthy gut microbiome composition, a high level of microbial diversity is certainly important. After treating mice with *A. muciniphila*, the α-diversity of their fecal microbiome was found to increase, as measured by Shannon’s diversity index, Ace, and Chao1 (Bian et al., 2019; Kim et al., 2021; Rao et al., 2021). These results imply that *A. muciniphila* can improve the richness and diversity of gut microbiota, which is a prerequisite for intestinal stability.

*Firmicutes* and *Bacteroidetes* are the two most abundant phyla in the human intestine (Qin et al., 2010). Human obesity is thought to be associated with an increased ratio of *Firmicutes*/*Bacteroidetes* (Magne et al., 2020), which may be due to differences in energy metabolism and inflammatory response (Krajmalnik-Brown et al., 2012; Bian et al., 2019). Notably, *A. muciniphila* was found to rescue mice with diabetes and NAFLD from *Firmicutes*/*Bacteroidetes* imbalance (Hanninen et al., 2018; Perez-Monter et al., 2022). *Firmicutes* and *Bacteroidetes* also differ in their SCFA profiles but may not be involved in the regulation of SCFA by *A. muciniphila* (Magne et al., 2020). In addition, the abundance of certain potential probiotics, such as *Lactobacillus* and *Verrucomicrobia*, has increased upon colonization with *A. muciniphila* (Bian et al., 2019; Xia et al., 2022).

However, the changes in intestinal microbiota following *A. muciniphila* administration reported in the current articles were not identical. For example, Hanninen et al. (2018) found that 4-week treatment with oral *A. muciniphila* reduced the abundance of Ruminococcus, while Bian et al. (2019) drew an opposite conclusion that the relative abundance of Ruminococcus was increased after *A. muciniphila* treatment compared with the dextran sulfate sodium-treated group. This may be due to the different sample sources for microbiota analysis, Hanninen et al. (2018) isolated bacterial DNA from stools or caecal and colon contents, while Bian et al. (2019) extracted bacterial DNA from fecal samples only. Different frequency and periodicity of *A. muciniphila* administration also influenced the results. At present, the low reproducibility and high bias of microbial sequencing results may be explained by methodological differences in sample sources and sequencing technologies, as well as the lack of consideration of environmental factors (Cani, 2018). Notably, *A. muciniphila* is a crucial regulator of the gut microbiota balance, and its precise role in the intestinal ecosystem remains to be explored.

### 4.4. Components of *Akkermansia muciniphila* active against T2D

Culture conditions can affect the potency of probiotics, live and heat-killed bacteria may differ in functions. Recent studies have focused on the active components of *A. muciniphila*. Heat-killed *A. muciniphila* is generally considered ineffective (Everard et al., 2013). Contrarily, many studies have argued that pasteurized *A. muciniphila* is equivalent to live *A. muciniphila* in providing metabolic improvement (Plovier et al., 2017; Depommier et al., 2020), and inflammation relief in obese and diabetic mice (Choi et al., 2021). Furthermore, pasteurized *A. muciniphila* has displayed increased effectiveness over the live microorganism in certain studies (Ashrafian et al., 2021). Notably, a recent study reported the anti-fibrotic properties of heat-killed *A. muciniphila* in human LX-2 cells, suggesting a possible role in liver fibrosis (Keshavarz Azizi Raftar et al., 2021). The above experiments indicate that certain critical substances from *A. muciniphila* are insensitive to heat and remain active after pasteurization or heating at 95°C. To determine the key components, Ottman et al. (2016) identified 79 outer membrane proteins of *A. muciniphila* using proteomics. Among these, Amuc_1100, encoded by the type IV pili gene cluster, is one of the most abundant proteins. Many studies have suggested that Amuc_1100 could partially recapitulate the beneficial effects of *A. muciniphila* in mice with diabetes or colitis (Plovier et al., 2017; Ottman et al., 2017b; Wang et al., 2020). Therefore, scientists have explored the underlying mechanism of Amuc_1100 functions, such as the activation of AhR (Gu et al., 2021), Toll-like receptor 2 (TLR2), and TLR4 (Plovier et al., 2017; Ottman et al., 2017b; Wang et al., 2021), reduction of colonic cytotoxic T lymphocytes (Wang et al., 2020), and inhibition of lipid synthesis and transport genes (Zhang F. L. et al., 2021), thereby alleviating T2D and related diseases. A recent study identified a lipid from *A. muciniphila*’s cell membrane as the main role of regulating immune homeostasis and dissected its structure and the mechanism of binding to toll-like receptors (Bae et al., 2022).

In addition, bacterial extracellular vesicles (EV) mediate signal transmission between the gut microbiota and the host (Villard et al., 2021). *A. muciniphila*-derived EVs (AmEV) play a significant role in improving the intestinal barrier (Chelakkot et al., 2018; Ashrafian et al., 2019), and regulating serotonin levels (Yaghoubfar et al., 2020). Thus, AmEV exhibits anti-inflammatory action and metabolic regulation. In detail, AmEV relieves inflammation by strengthening tight junctions and regulates metabolic imbalance by improving glucose tolerance (Chelakkot et al., 2018). In summary, AmEV and the outer membrane protein Amuc_1100 possess partial functions of *A. muciniphila*; however, further experimental investigations are needed to evaluate their potency and full potential as therapeutic agents.

## 5. Cultivation and measures to enrich the intestinal abundance of *Akkermansia muciniphila*

The ameliorative effects and mechanisms of *A. muciniphila* on T2D and related metabolic diseases have been described previously, which remind us that *A. muciniphila* is a promising probiotic and that oral administration of *A. muciniphila* may alleviate metabolic disorders. The method of cultivation and administration of *A. muciniphila* can greatly affect its function. Owing to the potential of *A. muciniphila* in treating metabolic diseases, many researchers have explored the most suitable growing environment and method for *A. muciniphila* in the intestine. Suitable culture conditions will allow *A. muciniphila* to survive better so that they can be administered by gavage to observe their effect on metabolic diseases. When *A. muciniphila* was first discovered by Derrien et al. (2004) this bacterium was defined as strictly anaerobic and grew well on mucin medium, with an optimal pH of 6.5 and growth temperature of 37°C. However, later studies suggested that *A. muciniphila* could grow under a low oxygen concentration (Ouwerkerk et al., 2016; Machado et al., 2020). Ottman et al. performed a transcriptomic and proteomic analysis on *A. muciniphila* cultured in mucus and non-mucus sugars. The results showed that several mucin-derived monosaccharides could be utilized by *A. muciniphila*. Furthermore, the addition of mucin enhanced the uptake of monosaccharides and the growth rate of *A. muciniphila* (Ottman et al., 2017a). The investigators also demonstrated that hexosamines, N-acetylgalactosamine (GalNAc), and N-acetylglucosamine (GlcNAc) served as critical nitrogen sources in the mixed sugar medium; Plovier’s discovery also supported this finding. Notably, the researchers found that the *A. muciniphila* cultured in a medium supplemented with glucose, GlcNAc, soy peptone, and threonine had the same growth efficiency as that in a mucus-based medium (Plovier et al., 2017). Additionally, a recent study reported that only when the concentration of mucin reached 0.5% m/v in the medium could the metabolic characteristics of *A. muciniphila* be altered (Liu X et al., 2021).

Oral drugs need to pass through the stomach to reach the intestine, so to achieve the purpose of drug delivery, *A. muciniphila* should be delivered in a way that maintains their activity as much as possible. However, probiotic delivery methods require improvement. Considering the characteristics of *A. muciniphila* and the gastric oxygen-rich and acidic environment, researchers have attempted to encapsulate live *A. muciniphila* in a water-in-oil-in-water double emulsion (van der Ark et al., 2017), freeze-dried xanthan, gellan gum matrix (Marcial-Coba et al., 2018), and spray-dried modified sodium alginate (Chang et al., 2020). *A. muciniphila* microencapsulation reinforced its activity and survival rate in gastrointestinal transit. Further research on microencapsulation would be of great help in improving the next-generation probiotics. In addition to direct *A. muciniphila* gavage, the intake of multiple dietary components and medicines, classified as polyphenols, flavonoids, probiotics, alkaloids, etc., could increase the abundance of *A. muciniphila* (Table 2).

**Table 2:** Dietary interventions that increased the abundance of *A. muciniphila.*

Intervention	Specific classification	Species	References
Polyphenols	Grape extract	Mice	Han et al. (2020), Lu et al. (2021), Roopchand et al. (2015)
	Berry extract		Anhe et al. (2015, 2018)
	Apple extract		Li et al. (2021), Liu F et al. (2021), Liu Q et al. (2021), Liu X et al. (2021)
	Green tea extract/Epigallocatechin-3-gallate		Jeong et al. (2020), Liu JH et al. (2020), Liu X et al. (2020), Sheng et al. (2018)
	Pomegranate extract	Human	Henning et al. (2017)
	Whole grape powder		Yang et al. (2021)
Saccharides	Inulin	Mice	Li T et al. (2022), Perez-Monter et al. (2022)
	Plant polysaccharide		Chang et al. (2015), Chen et al. (2021), Shang et al. (2017, 2018), Yde et al. (2021), Zhang et al. (2022)
	Dietary fiber		Bang et al. (2019), Tian et al. (2021)
	Oligosaccharide		Deng et al. (2021), Xi et al. (2020)
	Inulin	Human	Roshanravan et al. (2017)
	Dietary fiber		Zhang et al. (2019)
Probiotics	Lactobacillus	Mice/Rat	Ma et al. (2020), Ondee et al. (2021)
	Tibet kefir milk		Gao et al. (2021)
Flavonoids		Mice	Bu et al. (2021), Dong et al. (2021), Duan et al. (2021)
Alkaloids	Plant alkaloids	Mice	Shen et al. (2017). Yu et al. (2021)
	Breast milk alkaloids		Ribo et al. (2021)
Time-restricted feeding	/	Rat	Palomba et al. (2021)
Traditional chinese medicine	/	Mice/Rat	Li X et al. (2022), Regnier et al. (2020), Su, 2020, Wang et al. (2015, 2019), Zhai et al. (2021), Zhu et al. (2018)

## 6. Conclusions and perspective

Ever since it was isolated, *A. muciniphila* has been considered a therapeutic target for metabolic diseases, especially T2D. Although accumulating evidence has shown the positive effects of *A. muciniphila* on T2D and related diseases, the underlying mechanisms remain obscure. In this review, we focused on the mechanisms by which *A. muciniphila* ameliorates T2D and its potential as a next-generation probiotic. However, certain problems remain to be addressed. First, most controlled experiments were conducted using experimental animals as hosts; thus, more large-scale clinical studies should be performed to validate the function of *A. muciniphila* in humans. Second, the reproducibility of microbial gene sequencing has been complicated by environmental factors and methodological divergences. Therefore, sampling from both stool samples and different parts of the intestine may be necessary to ensure the consistency of the experimental findings. Furthermore, it would make more sense to focus on the microbial dynamic changes associated with *A. muciniphila* administration rather than sampling at a fixed time. Third, the discovery of *A. muciniphila*-derived substances provides a step forward in identifying potential therapeutics, and it is necessary to evaluate their bioequivalences with *A. muciniphila* itself. Finally, considering the heterogeneity of diabetes, individualized research and treatment options for microbiota are essential.

## Author contributions

YX and XJ: conceptualization, validation, writing—review and editing, and funding acquisition. JL: methodology, formal analysis, and writing—original draft preparation. QZ: software. GY: investigation. YX: resources and project administration. ZL, data curation. XJ: visualization and supervision. All authors contributed to the article and approved the submitted version.

## Funding

This research was funded by the National Natural Science Foundation of China (No. 82170369), the Jilin Provincial Science and Technology Foundation (Nos. 20210509003RQ and 20210402002GH), and the Changchun Science and Technology Bureau Development Plan project (No. 21ZY29).

## Conflict of interest

The authors declare that the research was conducted in the absence of any commercial or financial relationships that could be construed as a potential conflict of interest.

## Publisher’s note

All claims expressed in this article are solely those of the authors and do not necessarily represent those of their affiliated organizations, or those of the publisher, the editors and the reviewers. Any product that may be evaluated in this article, or claim that may be made by its manufacturer, is not guaranteed or endorsed by the publisher.
